# Arthroscopic Suprapectoral Biceps Tenodesis Using an Onlay Technique

**DOI:** 10.1016/j.eats.2024.103123

**Published:** 2024-07-02

**Authors:** Kenneth Cutbush, Kathir Azhagan Stalin, Helen Ingoe, Roberto Pareyón, Brandon Ziegenfuss, Ashish Gupta

**Affiliations:** a Queensland Unit for Advanced Shoulder Research (QUASR), Queensland University of Technology, Brisbane, Australia; b School of Surgery, University of Queensland, Brisbane, Australia; c Kenneth Cutbush Shoulder Clinic, Brisbane, Australia; d Australian Shoulder Research Institute, Brisbane, Australia; e Greenslopes Private Hospital, Brisbane, Australia

## Abstract

Tenodesis of the long head of biceps is a common shoulder surgical procedure. Tenodesis can be performed either arthroscopically or open and within the glenohumeral joint, within the bicipital groove, or below the pectoralis major tendon insertion. Arthroscopic tenodesis of the biceps tendon reduces the risk of infection. Our technique may also address persistent pain due to over tensioning of the tenodesis or from lesions hidden within the groove, such as bicipital synovitis or partial tendon tears, that are not visualized in a standard open technique. We describe an all-arthroscopic onlay technique for biceps tendon fixation at an extra-articular position within the bicipital groove, above the pectoralis major insertion. The technique uses standard arthroscopic equipment and a single knotless suture anchor.

There are multiple indications for long head biceps tenodesis (LHBT), including intrinsic and extrinsic biceps pathologies or as part of a subscapularis tendon repair.^1^ It is preferred in young individuals and typically achieves more cosmetically appealing results compared with tenotomy.^2^ Arthroscopic approaches may minimize short-term complications associated with open techniques, including infection.^3^ Our technique may also address persistent pain due to overtensioning of the tenodesis or hidden lesions within the groove, such as bicipital synovitis or partial tendon tears, that are not visualized in a standard open technique.^4^

We describe a knotless, all arthroscopic extra-articular and suprapectoral LHBT using an onlay technique in the bicipital groove.

## Surgical Technique

The technique is demonstrated in Video 1 and summarized in Table 1.

**Table 1:** Steps of the Procedure

Perform the diagnostic arthroscopy to identify the concomitant lesions
Visualize the biceps tendon from C portal
Establish the biceps working J portal
Identify and expose the long head of biceps in the bicipital groove
Establish the working L portal for placement of the luggage loop
Use the fiber link suture to make the luggage loop configuration around the tendon using KingFisher and bird-beak penetrator
Prepare the fixation site and make a pilot punch
Fix the fiber link suture to the bone using the SwiveLock suture anchor
Release the tendon at its proximal origin, and resect the tendon stump

**Fig 1:**
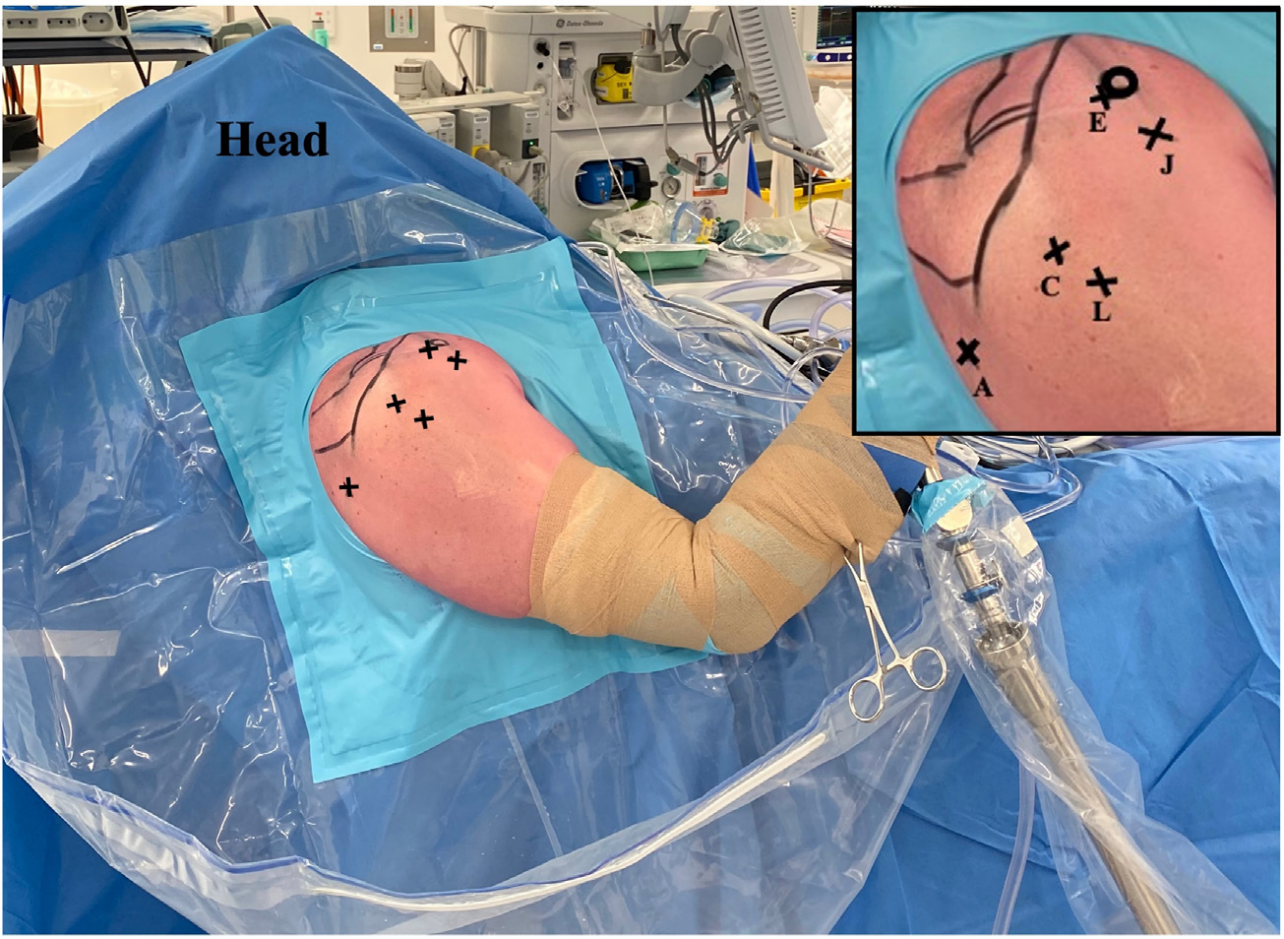
Patient positioned in the beach chair position with the Tmax table and spider at 20° forward flexion and in longitudinal traction. The patient’s right shoulder is shown with the ports used for the tenodesis marked. “A” is the standard viewing portal, “C” is the working portal for subacromial decompression and viewing portal for long head biceps tenodesis, and “J” working portal to identify and prepare the bicipital groove and to pass the anchor. “L” is the working portal to pass the suture and create the loop.

After general anesthesia, the patient is positioned in the beach chair position using the Tmax Shoulder Positioner (Smith & Nephew). The shoulder is examined under anesthesia to assess stability. Thereafter, the patient’s arm is held and supported by the Spider Limb II Positioner (Smith & Nephew) at 20° forward flexion and in longitudinal traction. Figure 1 demonstrates the limb position and the portal incisions. Portal descriptions are based on a previously published technique.^5^

Shoulder arthroscopy is performed using a 30° arthroscope from the posterior A portal. The long head of the biceps tendon is swept into the glenohumeral joint space using a 14-gauge Jelco needle through the rotator interval (portal E) for intra-articular inspection. The subacromial space is entered through the A portal. The C portal is created lateral to the acromion and 1 cm posterior to the anterior margin of the acromion using an outside-in technique marking the position and direction with a needle. A subacromial bursectomy is performed as required, and the arthroscope is transferred to the lateral C portal.

**Fig 2:**
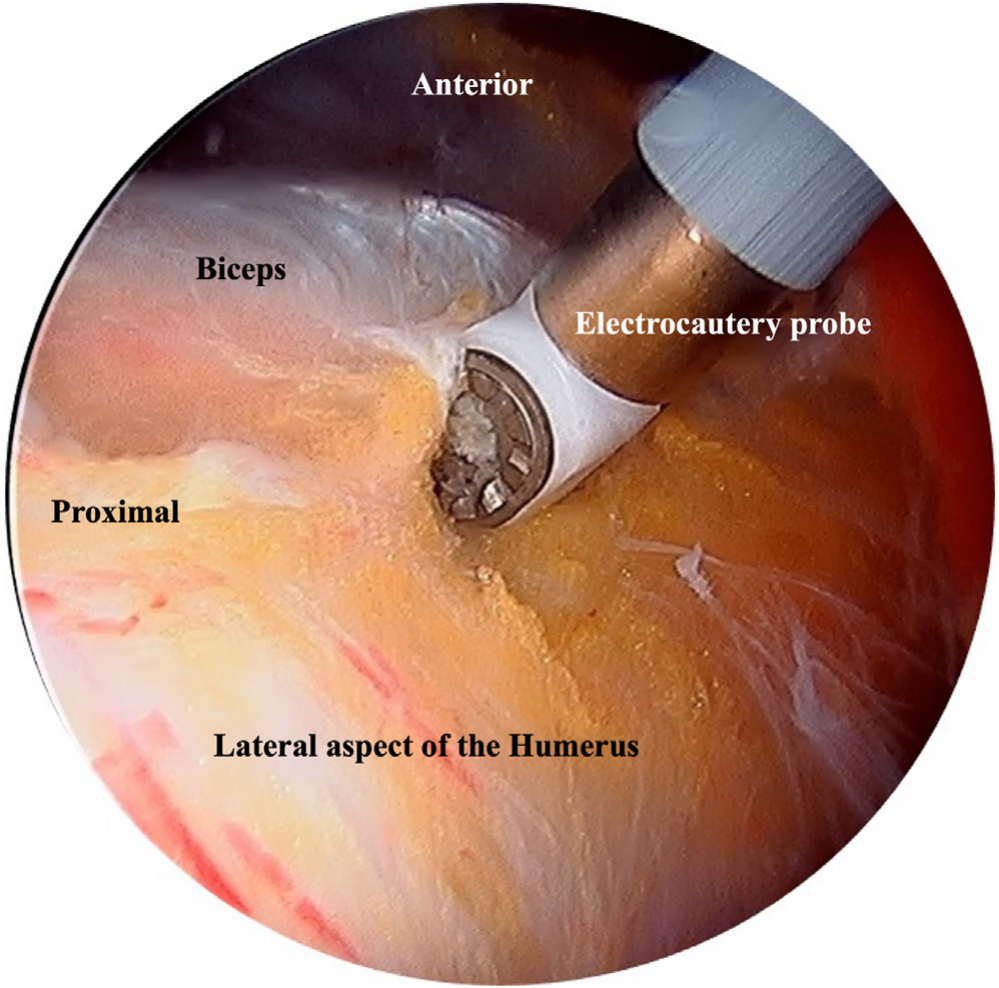
An arthroscopic image depicting the wave test used to locate the biceps tendon. The patient is positioned in the beach-chair position with the Tmax table and spider at 20° forward flexion and in longitudinal traction. The patient's right shoulder is shown. View is from a “C” portal lateral to the acromion and 1 cm posterior to the anterior margin of the acromion. The tip of the electrocautery probe, introduced through a “J” portal over the midportion of the bicipital groove, is placed in contact with the bone of the lateral aspect of the proximal humerus and then swept forward, watching for the wave effect to identify the bicipital groove.

A working J portal is created over the midportion of the bicipital groove using an outside-in technique. The soft tissue shaver is introduced through this portal, and soft tissue obscuring the bicipital groove is debrided. The position of the bicipital groove and the tendon is confirmed using the wave test, where the tip of the shaver or electrocautery is placed in contact with the bone of the lateral aspect of the proximal humerus and then swept forward to identify the bicipital groove (Fig 2). As the tip rolls across the bicipital groove, a wave effect is observed. This wave effect is not observed over the bone of the proximal humerus or the subscapularis tendon anteriorly. Visualization and access to the bicipital groove can be improved by increasing the degree of arm forward flexion. The tip of the shaver probe is positioned directly over the midportion of the bicipital groove, and pressure is applied while the probe tip is swept up and down to open the fascia lying over the bicipital groove. The long head of the biceps tendon can be lifted out of the groove with the shaver and examined. The electrocautery probe is used to open the fascia further over the groove and control any bleeding vessels.

**Fig 3:**
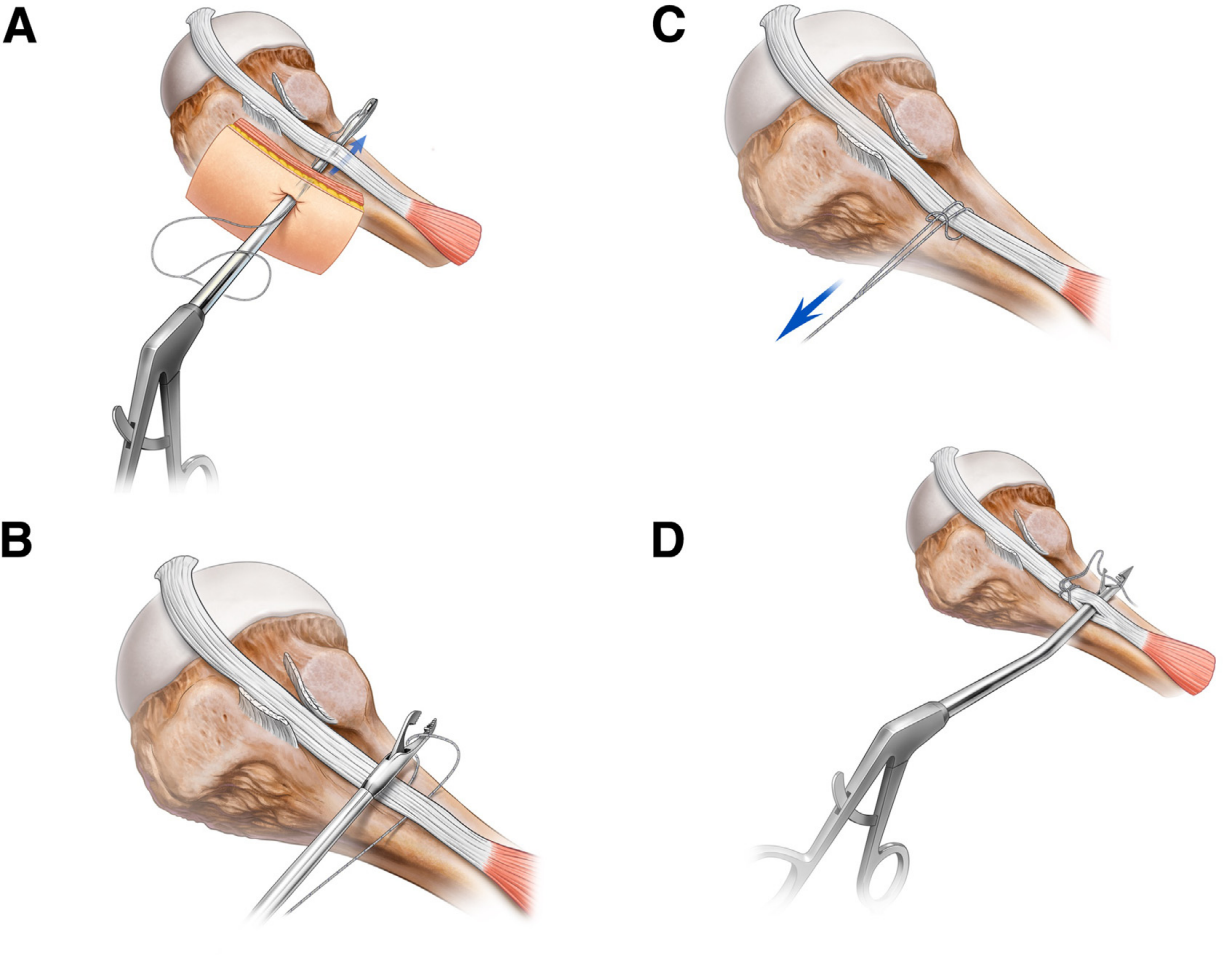
The patient is positioned in the beach-chair position with the Tmax table and spider at 20° forward flexion and in longitudinal traction. The patient’s right shoulder is shown. (A) A Kingfisher loaded with 1.7-mm loop suture tape, introduced through the “L” working potal, is passed under the biceps tendon. (B) Working from the “L” portal, the grasper is then passed forward over the biceps tendon to retrieve the tail end of the loop suture tape out of the shoulder, while (C) cinching the loop down to the tendon. (D) A bird-beak penetrator, introduced through the “L” portal, retrieves the suture tape tail through the penetrated tendon.

Another small L working portal is created 1.5 cm below and slightly posterior to the C portal where the arthroscope is sitting. A suture tape with a fixed loop at one end (1.7 mm Suture Tape, AR-7538T Arthrex TigerLink, Arthrex, Naples, FL) is loaded onto an arthroscopic grasper (KingFisher Suture Retriever/Tissue Grasper with SR Handle AR-13970SR, Arthrex). The loop end of the suture tape is threaded over the shaft of the grasper, and the suture tape tail is grasped in its tip (Fig 3A). This grasper is introduced into the L portal, then passed forward in the subacromial space and under the biceps tendon (Fig 3A). The tail of the loop suture tape is placed in the anterior part of the subacromial bursa. The grasper is then withdrawn from under the biceps tendon until it sits immediately posterior to the biceps tendon. The grasper is then passed forward over the biceps tendon to retrieve the end of the suture tape, which had earlier been placed anterior to the biceps tendon (Fig 3B). The suture tape end is held in the grasper while it is retrieved out of the shoulder through the L portal. The retrieved loop end of the suture tape is cinched down along its tail until the biceps tendon is grasped in a luggage hitch (Fig 3C).

**Fig 4:**
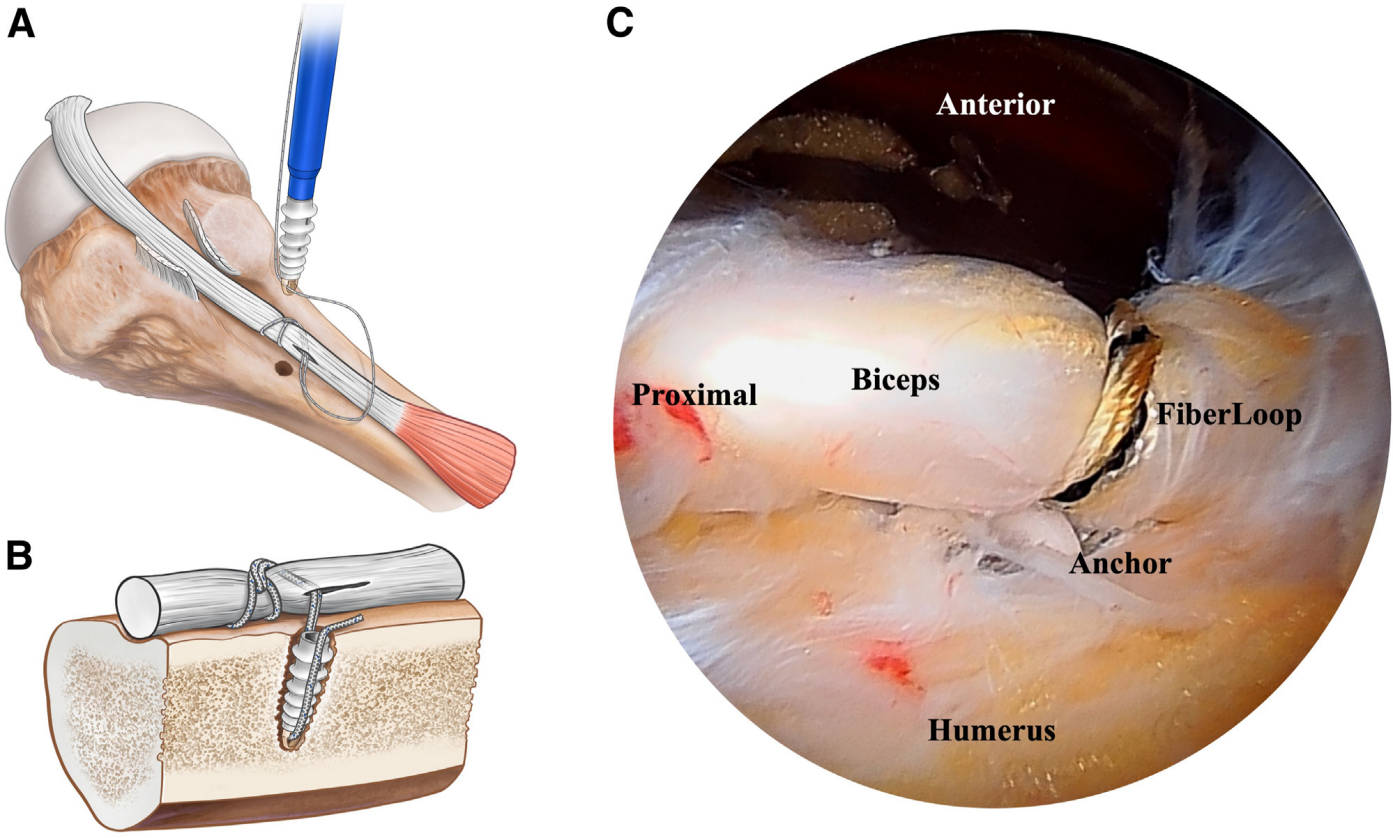
The patient is positioned in the beach-chair position with the Tmax table and spider at 20° forward flexion and in longitudinal traction. The patient’s right shoulder is shown. (A) The tail end of the suture tape is loaded into the anchor. A pilot hole is created with a bone punch adjacent to the hitch in the biceps tendon. (B) A knotless suture anchor is inserted into bone. (C) Arthroscopic view of the final construct looking from posterior to anterior.

Using the suture grasper either through the L or J portal, the suture tape adjacent to the looped hitch is placed anterior to the biceps tendon and distal to the cinch for subsequent retrieval. A bird beak penetrator (Penetrator FiberTape Retriever, 15° with SR Handle, Up Curved, AR-2167-3, Arthrex) is passed from the L portal through the biceps tendon distal to the looped hitch (Fig 3D). The suture tape tail is grasped with the penetrator and retrieved through the biceps tendon, locking the hitch. A pilot hole is created with a bone punch (AR-1927PB, Arthrex) adjacent to the hitch in the biceps tendon through the J portal. The tail end of the suture tape is then loaded into the anchor (BioComposite SwiveLock C, 5.5 mm × 19.1 mm, AR-2323BCC, Arthrex) and the anchor deployed, securing the biceps tenodesis (Fig 4A and B). A suture cutter (Mini Suture Cutter, 3.4 mm, Straight AR-13255, Arthrex) is used to cut the suture tail to length (Fig 4C).

With the tenodesis completed the arthroscope is replaced into the glenohumeral joint through the A portal. The biceps tendon is divided at its insertion to the superior labrum with a straight arthroscopic punch (Capsular Release Punch; ACUFEX; Smith & Nephew) introduced through the E portal lateral to the coracoid marking. The proximal long head of the biceps tendon stump is trimmed using the soft tissue shaver. Incisions are closed with adhesive steristrips (Steri Strip, 3M).

Postoperatively, patients are immobilized in a sling for 6 weeks and advised not to perform any active elbow flexion. At 6 weeks postoperatively, the sling is removed, and patients are allowed to perform active elbow flexion without weight. At 3 months postoperatively, progressive strengthening is commenced. Patients are advised not to perform a maximal lift with the arm until 6 months postoperatively.

## Discussion

This all-arthroscopic knotless extra-articular method is a safe, reproducible technique for LHBT in the bicipital groove above the pectoralis major tendon insertion. Advantages and disadvantages of the technique are described in Table 2. Pearls and pitfalls are described in Table 3.

**Table 2:** Advantages and Disadvantages

Advantages
• Relatively safe and reproducible onlay technique
• Facilitates identification of intra-articular and bicipital groove lesions
• Exteriorization of the tendon is not required, thereby reducing the risk of infection
• Facilitates maintenance of correct length-tension relationship of the tendon
• Reduces risk of fracture compared with some interference techniques
• Knotless technique ensures that knot-related complications to soft tissues are avoided
• Fast technique once mastered
Disadvantages
• Learning curve for beginners
• Potential fixation failure because it is heavily reliant on anchors until fibrous healing
• Possibility of suture pull-out
• Cost
• Six weeks in sling

**Table 3:** Pearls and Pitfalls

Pearls
• Use the wave test to identify the biceps tendon in the bicipital groove
• Forward flexion of the arm will often improve visualization of the operative site
• Place the L working portal slightly more posterior than the C portal used for arthroscopic visualization
Pitfalls
• Locating the biceps tendon in the bicipital groove can be difficult
• Bleeding can often occur when the fascia is opened over the bicipital groove
• Must remember to release the biceps tendon from its labral insertion
• Avoid excessive stump resection because it may cause suture pull-out

An alternative to the onlay technique is an interference screw. Proposed advantages include increasing the contact area of tendon to bone and therefore healing potential in the suprapectoral region. Unfortunately, this has not been proven, with little evidence to suggest healing within the tunnel compared with the cortical surface.^6^ Compared with interference methods, the onlay technique also reduces the chance of compression damage of the tendon against the cortical bone at the site of screw tendon interface.^7^

Interference techniques are also prone to changing the length-tension relationship of the biceps tendon^8^ due to difficulties in accurately determining the length of tendon buried in the proximal humerus with the screw. Some authors suggest that overtightening the LHBT may cause persistent postoperative pain and predispose fixation failure.^8^ Another criticism of interference screw techniques is that they may increase the risk of fracture, requiring larger bone tunnels compared with 5.5-mm anchors.^9^ Notably, Patzer et al. found no statistically significant difference in strength between interference screws and suture-anchor constructs as a cause of early failure.^10^

Another potential cause for persistent postoperative pain is tendon lesions not appreciated at the time of tenodesis. The technique we describe requires the examination of the long head of the biceps tendon in its intra-articular and bicipital groove portions, thereby reducing the risk of missing concomitant pathologies.

Compared with subpectoral methods, our technique is a simple arthroscopic procedure and has several advantages. Subpectoral LHBT techniques are more technically challenging when performed arthroscopically. Whereas the primary subpectoral biceps tenodesis nets reliable clinical results with a relatively low risk of failure,^11^^,^^12^ subpectoral methods do not always adequately restore the length-tension relationship of the muscle.^13^

To conclude, the technique described is safe, simple, reproducible, and reduces risk of complications related to open subpectoral tenodesis and arthroscopic intra-articular biceps tendon fixation.

## Disclosures

The authors declare the following financial interests/personal relationships which may be considered as potential competing interests: K.C. reports a relationship with Stryker that includes consulting or advisory; a relationship with Arthrex Inc. that includes consulting or advisory; a relationship with Johnson & Johnson that includes consulting or advisory; a relationship with Arthrex that includes funding grants; a relationship with Stryker that includes funding grants and a relationship with Device Technologies that includes funding grants. A.G. reports a relationship with Akunah Medical Technology Pty Ltd that includes board membership; reports a relationship with Australian Shoulder Research Institute that includes funding grants; a relationship with Zimmer Biomet that includes consulting or advisory and travel reimbursement; a relationship with Queensland Unit for Advanced Shoulder Research that includes board membership, funding grants, and nonfinancial support; and previous consultancy work for Stryker. All other authors (K.A., H.I., R.P., B.Z.) declare that they have no known competing financial interests or personal relationships that could have appeared to influence the work reported in this paper.

## Supplementary Data

Video 1 In this video, we demonstrate an all-arthroscopic technique to fix the biceps tendon at an extra-articular position within the bicipital groove, above the pectoralis major insertion. The patient is in the beach-chair position with the arm held in a Spider II limb-positioning device at 20° forward flexion and in longitudinal traction. The right shoulder is shown, and the video begins with the arthroscope in the A viewing portal. The tendon is identified using the wave test and lifted out of the groove and examined. The fixation comprises a luggage loop configuration around the tendon using the TigerLink suture tape and secured to the bone using SwiveLock suture anchor. Portal key: “A” is the standard viewing portal, “C” is the working portal for subacromial decompression and viewing portal for long head biceps tenodesis, “J” is the working portal to identify and prepare the bicipital groove and pass the anchor, and “L” is the working portal to pass the suture and create the loop.
